# An Overview to the Health Benefits of Seaweeds Consumption

**DOI:** 10.3390/md19060341

**Published:** 2021-06-15

**Authors:** Silvia Lomartire, João Carlos Marques, Ana M. M. Gonçalves

**Affiliations:** 1 University of Coimbra, MARE-Marine and Environmental Sciences Centre, Department of Life Sciences, Calçada Martim de Freitas, 3000-456 Coimbra, Portugal; silvia.lomartire@uc.pt (S.L.); jcmimar@ci.uc.pt (J.C.M.); 2 Department of Biology and CESAM, University of Aveiro, 3810-193 Aveiro, Portugal

**Keywords:** seaweeds, bioactive compounds, human health, pharmaceutical application, nutraceutical application

## Abstract

Currently, seaweeds are gaining major attention due to the benefits they give to our health. Recent studies demonstrate the high nutritional value of seaweeds and the powerful properties that seaweeds’ bioactive compounds provide. Species of class Phaeophyceae, phylum Rhodophyta and Chlorophyta possess unique compounds with several properties that are potential allies of our health, which make them valuable compounds to be involved in biotechnological applications. In this review, the health benefits given by consumption of seaweeds as whole food or by assumption of bioactive compounds trough natural drugs are highlighted. The use of seaweeds in agriculture is also highlighted, as they assure soils and crops free from chemicals; thus, it is advantageous for our health. The addition of seaweed extracts in food, nutraceutical, pharmaceutical and industrial companies will enhance the production and consumption/usage of seaweed-based products. Therefore, there is the need to implement the research on seaweeds, with the aim to identify more bioactive compounds, which may assure benefits to human and animal health.

## 1. Introduction

Seaweeds are considered a nutrient-rich food as they are a good source of minerals, vitamins (A, B_1_, B_2_, B_9_, B_12_, C, D, E, and K), essential minerals (calcium, iron, iodine, magnesium, phosphorus, potassium, zinc, copper, manganese, selenium, and fluoride), dietary fibers [1,2,3,4], protein, essential amino acids and polyphenols, which exhibit antioxidant and anti-inflammatory properties [5]. Seaweeds possess a low lipid content, nonetheless enriched in polyunsaturated fatty acids. This characteristic makes them even more attractive, as they are a healthy, nutritive and low-caloric food [2]. Seaweeds were consumed as whole food since ancient times, and they still have great economic importance. *Saccharina* spp. with *Porphyra* spp. and *Undaria pinnatifida* (Phaeophyceae) are the three algae mainly consumed in Asian meals [6].

Seaweed bioactive compounds are also employed in biomedical and pharmaceutical industries as they possess antitumoral activity against some type of cancer cell lines, but they do not affect negatively healthy cells, as it happens with current antitumoral treatments [7,8]. Phycocolloids, which derive from brown and red algae, are used in the food industry (gelling agents), pharmaceuticals (dressings, coatings of medicaments) and biotechnology (culture medium, the Petri dishes). They are also found in cosmetics (body lotions, soaps, shampoos, toothpaste) [9]. Marine algae have been traditionally used in animal feed and in agriculture and production of biodiesel.

Seaweeds are classified as brown, red or green algae, and for each group are present diverse bioactive compounds with multiple properties which may be exploited for biotechnological applications. Phaeophyceae (brown algae) have been consumed as whole food for a long time in Asian countries; however, scientists have only recently gained an understanding of the reasons behind the positive effects that seaweed bioactive compounds have on our health. Brown algae possess fucoidans that are already available in the market as nutraceutical products, since they exhibit antibacterial [10], antiviral [11] anti-inflammatory, anticoagulant, and antithrombotic effects [12]. Rhodophyta (red algae) extracts are widely exploited in medical and pharmaceutical sectors, particularly carrageenans and agar. Agar is used in biomedicine as suspension component in drug solutions and in prescription products, but also as anticoagulant agents [13]. Carrageenans can be exploited for the production of antitumoral therapies, due to their antitumor immunity activation [7]. Chlorophyta (green algae) are rich in ulvan, a sulphate polysaccharide commonly used in biomedicine, cosmetic and pharmaceutical industries but also as emulsifiers, stabilizers, and thickeners in food products [14].

The human health benefits from seaweeds can be through direct and indirect way: through the consumption of the whole seaweed or the uptake by assumption of food supplement or natural drugs (direct health benefits) or by using seaweeds in agriculture as natural fertilizers, in order to have a nutrient soil and healthy cultivation without the presence of chemicals contained in traditional fertilizers. Thus, the use of seaweeds as biofertilizers will enhance the plants and soil conditions [15,16,17], giving positive effects to our health after agriculture products consumption (indirect health benefits).

In this review, the applications regarding seaweeds extracts are highlighted, with specific attention given to the health benefits of specific bioactive compounds. Therefore, further studies have to be performed in order to achieve deep knowledge about these powerful sources of nutrients and biological compounds.

## 2. Main Bioactive Compounds of Seaweeds

Seaweeds are rich in several bioactive compounds such as polyphenols, sterols, alkaloids, flavonoids, tannins, proteins with essential amino-acids, polyunsaturated fatty acids, etc. [13]. These bioactive compounds provide not only protection to seaweeds, but also a high nutritional value and several benefits for humans. For example, polysaccharides from seaweeds have a positive effect on intestinal tract, but contrary to fibres, they are free of calories [2]. Agar and carrageenan, extracted from red algae, and alginates, extracted from brown algae, are commonly employed in food and pharmaceutical products as stabilizers [18].

Due to their beneficial properties, these biological compounds extracted from marine algae have been received attention from researchers. These compounds might be employed for creation of novel and functional food but also for pharmaceutical and biomedical applications [19].

### 2.1. Phaeophyceae

The main compounds useful for biotechnological applications present in brown seaweeds are mentioned in Table 1.

**Figure 1:**
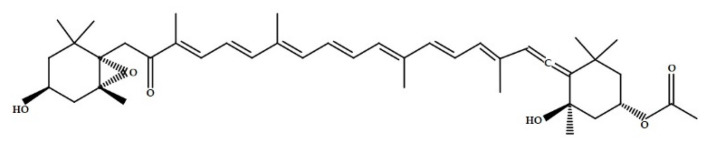
Chemical structure of fucoxanthin.

The typical brown colour of Phaeophyceae is due the presence of a particular pigment called fucoxanthin (Figure 1) [20,21], which structure varies among species [22]. Studies showed that fucoxanthins have antitumoral, antioxidant and anti-obesity properties [23,24,25].

In brown algae, laminaran and fucoidan are mostly interesting for their potential biological activities, while alginate is mostly used as food ingredients. In particular, fucoidans and laminarans contents may vary from species to species from 20% to 50% of algae dry weight [26].

**Figure 2:**
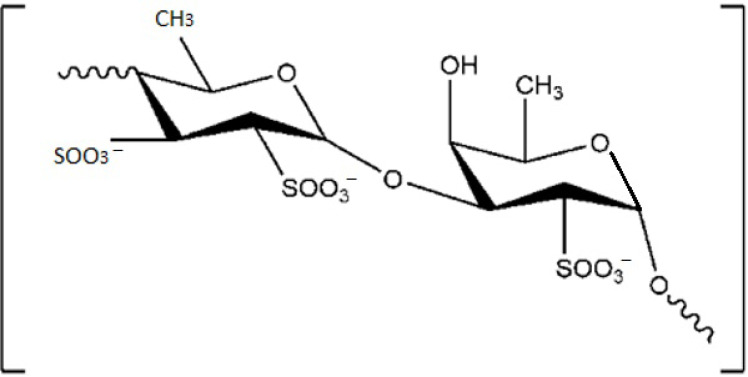
Chemical structure of sulphate polysaccharide (SP) fucoidan.

The sulphate polysaccharide (SP) fucoidan (Figure 2) presents a skeleton that is rich in fucose. This SP is present in high quantity; indeed, fucoidans may constitute up to 25–30% of the algae dry weight, depending on the seaweed species and on season [27,28]. The fucoidan structure is highly branched with specific sugars bonds [29,30,31] which can vary among species [30,32]. Fucoidans in seaweeds are indeed heterogenic; they present variations in terms of carbohydrate-unit content and content of sulphate and acetyl groups. Depending on the structure of the main chain, fucoidans may be sulphated at C4, C2 or both C2 and C4 positions of fucose units [28,33,34]. Some fucoidans may be both sulphated and acetylated [33,35]. The position and content of sulphate groups are important to the biological activities of SPs; thus, it is important to determine the structure of fucoidans in brown seaweeds. The methods of determining the sulphate position include infrared spectroscopy, desulfation, stability of sulphate esters to alkali and methylation analysis [30].

**Figure 3:**
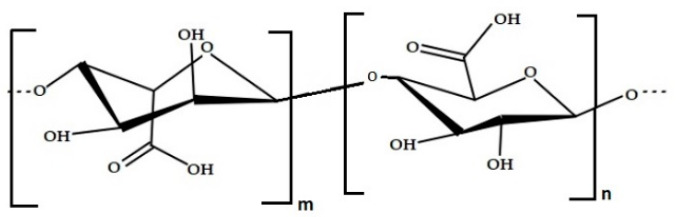
Chemical structure of alginic acid.

In terms of polysaccharides, the most specific one is the alginic acid, also called algin or alginate (Figure 3). These polysaccharides can be extracted from cell walls of brown algae including *Macrocystis pyrifera, Laminaria hyperborea, Ascophyllum nodosum,* and various bacterial strains [36]. Alginate can constitute between 10% and 40% of the dry weight (untreated) of the algae; 30–60% of the total sugars in brown seaweeds is composed by alginates [37]. The structure of alginic acid consists of a linear polysaccharide of 1,4-linked β-d-mannuronic acid (M) and 1,4 α-l-guluronic acid (G) residues arranged in homogenous (poly-G, poly-M) or heterogeneous (MG) block-like patterns [38]. Properties such as biodegradability, biocompatibility, non-toxic behaviour, and low cost would render alginates excellent candidates in biological applications [39]. Sources of alginates have been found in *Ascophyllum* spp., *Durvillaea* spp., *Laminaria* spp., *Lessonia* spp., *Macrocystis* spp*., Sargassum* spp. and *Ecklonia radiata* [40]. Fucales (large brown seaweed) also utilized for nutrition and alginates, include *Scytothalia dorycarpa*, *Cystophora subfarcinata* and *Sargassum linearifolium* [40].

**Figure 4:**
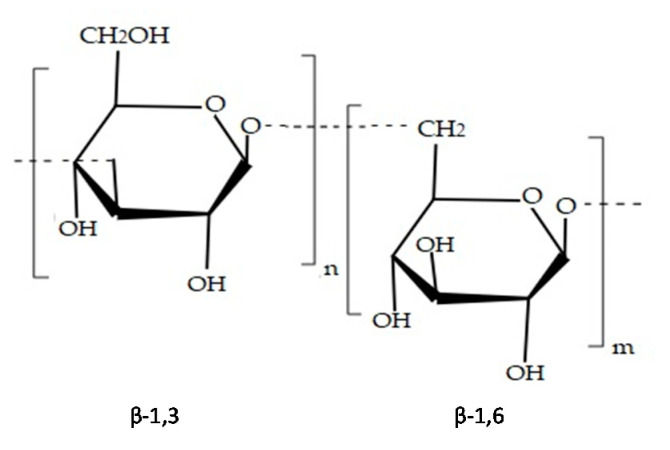
Chemical structure of laminaran.

Laminarans possess structures and composition that vary according to algae species [35]. These molecules are composed of (1,3)-β-d-glucan [26] with β-(1,6) branching [41] (Figure 4). There are two types of laminaran chains (M or G), which differ in their reducing end. M chains end with a mannitol residue, whereas G chains end with a glucose residue. Laminarans molecular weight is approximately 5000 Da. This molecule contains a high number of neutral sugars and low quantity of uronic acids. Presence of laminar molecules has been detected in *Saccharina longicruris, Ascophyllum nodosum* and *Fucus vesiculosus* [42].

Regarding polyunsaturated fatty acids (PUFAs), brown seaweeds are mainly composed of linoleic acid (C18:2*n*-6, LA), arachidonic acid (C20:4*n*-6, DHA) and eicosapentaenoic acid (C22:5*n*-3, EPA) [43,44]. Cholesterol is one of the major sterols presented in all groups of seaweed [45]. Besides that, brown and green algae are rich in other C29 sterols, particularly fucosterol and isofucosterol, respectively [46,47].

**Figure 5:**
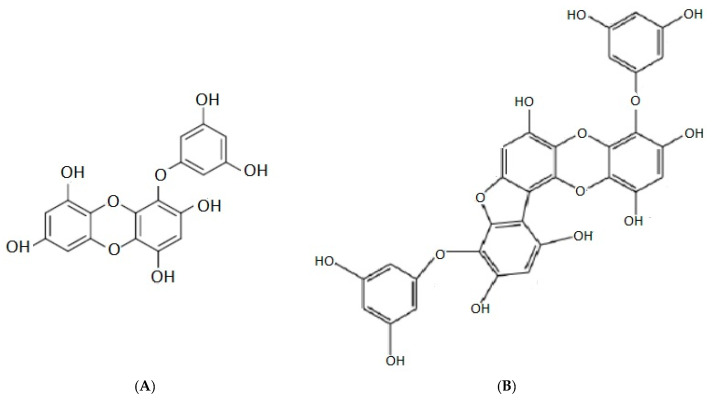
Chemical structure of eckol (**A**) and phlorofucofuroeckol A (**B**), phlorotannins isolated from *Ecklonia cava*.

Phlorotannins are very specific polyphenols present in brown algae. Their presence and activity in algae vary depending on species and environmental conditions. The biological functions of phlorotannins are to protect seaweeds from UV radiations, stress and herbivory but also to contribute to the cell wall resistance [48,49,50]. The structure of phlorotannin consist in phloroglucinol (1,3,5-trihydroxybenzene) units that are bonded to each other by different pathways. They are found in the range 126–650 kDa, and their concentration in dried brown seaweeds varies from 0.5% to 2.5% [51,52]. The biological activities exerted by phlorotannins are multiple, including anticancer [53] antioxidant [54], anti-inflammatory [55] antidiabetic, and neuroprotective [56] activities. This suggests that these compounds are potentially useful as new ingredients in food [57,58], animal feed, and pharmaceutical industries. Among brown seaweeds, *Ecklonia cava* has been reported to produce higher concentrations of phlorotannins than other marine phenolic compounds [59]. *Ecklonia cava* contains triphlorethol A, fucodiphlorethol G, dieckol, dioxinodehydroeckol, phloroglucinol eckol and phlorofucofuroeckol A (Figure 5A,B) [53], and they also exhibit antihypertensive [60] effects.

*Eisenia bicyclis*, *Eisenia arborea*, *Ecklonia cava*, *Ecklonia kurome*, *Ecklonia stolonifera*, *Pelvetia siliquosa*, and *Ishige okamurae* contain phlorotannins with antidiabetic, antioxidant, antitumor, anti-inflammatory, and anticancer properties [61]. Phlorofucofuroeckol A extracted from *Eisenia arborea* showed antiallergic effects [62].

### 2.2. Rhodophyta

Marine red algae produce high quantity of secondary metabolites [63], including sesquiterpenes, diterpenes, triterpenes and many other types that exert various biological activities such as antifungal, antibacterial, and anticancer activities [64].

The main compounds useful for biotechnological applications present in red seaweeds are mentioned in Table 2.

**Figure 6:**
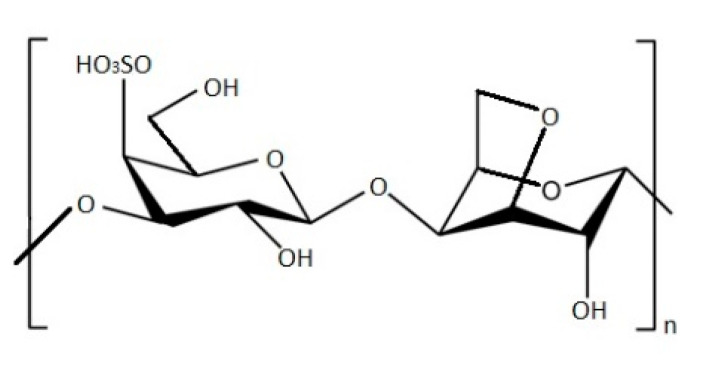
Chemical structure of κ-carrageenan.

Carrageenans are SPs present only in red algae [7]. Their structure is constituted by SPs (galactans), which can form gels in water or milk solutions. This phycocolloid is used mainly in food, cosmetic and pharmaceutical industry. Tests on carrageenans demonstrated that they express immune stimulating, antioxidant, anticoagulant antitumoral and antiviral properties [65,66], which make red algae extracts commercially exploited [13,67]. These natural polysaccharides are a mix of sulphated linear galactans and their structural units are presented with the disaccharides of α-(1→4)-linked d-galactopyranose (D) residue or 3,6-anhydrogalactopyranose (DA) and β-(1→3)-linked d-galactopyranose (G) residue. Sulphate groups are covalently linked via ether bonds to the carbohydrate atoms C-2, C-4 or C-6 of galactose [7]. Usually, κ-carrageenans (Figure 6) available in the market are extracted from *Kappaphycus alvarezii* using the hot extraction processing, while λ-carrageenans are commonly isolated from red algae belonging to the genera *Gigartina* or *Chondrus* with the use of drum dryer procedure or ethanol precipitation [68]. These SPs have been found in the families Solieriaceae, Rhabdoniaceae, Phyllophoraceae, Gigartinaceae, Rhodophilidaceae, and Thichocarpaceae. Eight sources of carrageenans were discovered exclusively in the Japanese sea (East Sea), and five of them (*Chondrus pinnulatus*, *Chondrus armatus*, *Chondrus yendoi*, *Mastocarpus pacificus*, *Mazzaella hemisphaerica*) belong to the Gigartinaceae and Solieriaceae. In addition to Gigartinaceae, high carrageenan contents were found in the algae of the families Phillophoraceae and Thichocarpaceae, species of which are widely spread in all far eastern seas [7].

**Figure 7:**
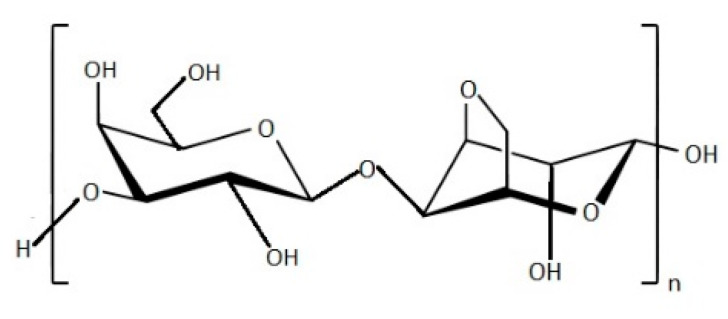
Chemical structure of agarose polymer.

Agar is a collective term used to describe a mixture of gelling polysaccharides made up of d-galactose and l-galactose [69]. This mixture is synthesized in the cell wall matrix of red seaweeds. It remains in the form of gel under ambient temperature [70]. The polysaccharide that presents repeated d-galactose and 3,6-anhydro-l-galactose units joined by β-1,3- and α-1,4-glycosidic bonds is commonly known as agarose (Figure 7) [71], which contribute up to 70% of agar polysaccharide.

Agar and other polysaccharides provide resistance to pathogens, maintain cellular ionic equilibrium, protect algae against extreme salinity, pH and temperature, and desiccation [9,72].

Moreover, diverse agars are implied in different applications as phycocolloids in food, pharmaceuticals, cosmetic, medical and biotechnology industries. Global production of agar has escalated from 6800 tons (USD 82.2 million) in 2002 to 9600 tons (USD 173 million) in 2009, with *Gracilaria* spp. (80%) and *Gelidium* spp. (20%) as the largest agar industrial sources [73], reported as the species with highest sulfation degree [74]. *Gracilaria* spp. agars with lower gel strength have gained more attention due to depleted *Gelidium* spp. stocks and the successful cultivation of *Gracilaria* [75,76].

### 2.3. Chlorophyta

The main compounds useful for biotechnological applications present in green seaweeds are mentioned in Table 3.

Chlorophyta present their typical green colour due to the presence of chlorophylls (a and b) and carotenoids (β-carotene and xanthophylls). Pigments in seaweeds are important because they possess antioxidant activity [77]; thus, they protect seaweeds against harmful effects experienced due to irradiance.

In terms of PUFAs, Chlorophyta are mostly composed by the C16 and C18 PUFA, namely the linoleic acid (LA; C18:2*n*-6) in most of the species. However, α-linolenic acid (ALA, C18:3*n*-3) is characteristic of Ulvales [78,79,80]. Contrasting with red and brown algae, green algae also contain large amounts of palmitic (16:3*n*-3; 16:4*n*-3)) PUFAs. Regarding the carbohydrates, Chlorophyta are rich in SPs that constitute the cell walls [81]. The PUFA content of the green seaweed *Ulva linza* exhibited the best inhibitory activities against inflammatory response [82]. Monounsaturated fatty acid (MUFA) derivates extracted from *Ulva lactuca* have been discovered to induce many antioxidant-response elements which release antioxidant genes in various mouse tissues [83]. Moreover, seaweeds such as *Codium* spp., *Ulva* spp. and *Chaetomorpha* spp. possess other interesting compounds, for example the sterols 28-isofucosterol [79,84], ergosterol and 24-ethylcholesterol [85]. Green seaweeds possess high content of polysaccharides, for example the 65% of dry weight of *Ulva* spp. contains polysaccharides [86].

**Figure 8:**
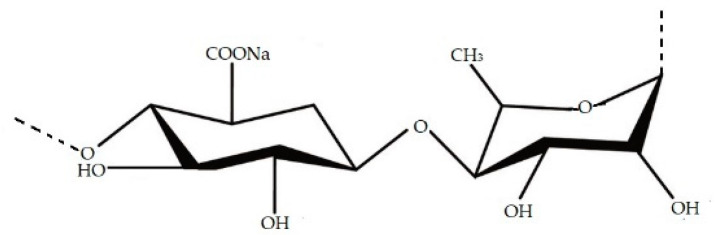
Chemical structure of ulvan.

The most important SP extracted from cell wall of green seaweeds is ulvan. This compound, with fucoidan and carrageenan, has wide application in many fields, for example, in cosmetics formulations to produce hair conditioners, moisturizers, emulsifiers, wound-healing agents, and as a thickening agent [9]. Ulvan is generally present for 9–36% dry weight of *Ulva* biomass [87,88]. The chemical structure of this SP is mainly composed of sulphated rhamnose, glucuronic acid, iduronic acid, and xylose and repeating disaccharide structure comprised of an uronic acid linked to a sulphated neutral sugar (Figure 8) [87]. Ulvan possesses antioxidant activities which makes it a potential candidate to be involved in diverse applications, such as pharmaceutical, agricultural, medical applications and production of alternative biomaterial [81,89].

## 3. The Health Benefits of Seaweed Bioactive Compounds

Seaweeds bioactive compounds are exploited in several biotechnological applications (Table 4). Due to their properties, these compounds can contribute to the development of biomedicine and modern pharmacy, in order to achieve new formulation based on components from natural origin. The consumption of seaweeds by food or trough natural drugs will contribute to the occurrence of a healthier lifestyle. Nutraceutical, biomedical and pharmaceutical applications that involve seaweeds’ bioactive compounds are further showed.

### 3.1. Nutraceutical Applications

It is identified as “nutraceutical food” a food that provides not only nutritional value but also it may help to prevent health problems. Seaweeds-based foods are considered nutraceutical products due to the positive effects on human health, for example, to alleviate arthritis, diabetes, autoimmune, ocular and cardiovascular diseases [102].

Phaeophyceae has been consumed as whole food for a long time in Asian countries. Currently, seaweed extracts are receiving more attention. Fucoidan is the main component of brown algae with a broad spectrum of action, the effect of which depends on various factors, including molecular weight, as Dörschmann and Klettner [111] highlighted. For example, the positive angiogenesis activity exerts by fucoidans mostly depend on the molecular weight of the fucoidan: with low-molecular-weight fucoidans are generally considered to be pro-angiogenic, while fucoidans with high-molecular-weight fucoidans are considered to be antiangiogenic [112]. High-molecular-weight fucoidans have been discovered to enhance the viability and prevents the death of spleen cells [113].

Fucoidans present in some brown seaweed species are commercially available as nutraceutical products in Australia and the USA, since they express several biological activities after the uptake, including antibacterial [10], antiviral [11] anti-inflammatory [12], anticoagulant, antithrombotic [12], antidiabetic [114], procoagulant [115], anticancer [116] and antiviral activities [117]. Currently, fucoidans can be considered promising molecules for biomedical application; therefore, they are still not approved for medical applications [118,119]. Tests were carried out to detect the anticoagulant activity of fucoidan. Fucoidans isolated from *Fucus vesiculosus* were injected on mice. After a single-dose oral administration, the anticoagulant activity was established with the anti-Xa assay, a laboratory test which indirectly measures the activity of heparins [120].

Moreover, the chemical diversity of fucoidan and its antioxidant capacity can contribute to the prevention of human diseases [12,121,122] such as cancer, diabetes, Alzheimer’s disease, Parkinson’s disease and AIDS [22,123,124]. For example, fucoidan from *Cladosiphon okamuranus* showed in vivo apoptotic activity in mice cancer cells enhancing anti-proliferative activity without any negative consequence of normal epithelial cells [125]. Fucoidans with low molecular weight extracted from *Lonicera japonica* exhibited reduced diabetic retinopathy during in vivo test, decreasing the high-glucose-induced proliferation in cells and the retinal neovascularization and retinal damage as well [126]. Trinchero et al. [127] tested fucoidans isolated from *Adenocystis utricularis* that showed in vitro antiviral property against HIV-1 trough prevention of HIV-1 replication. Phlorotannins are already available in the market as food supplements and functional food ingredients [57,58]. The European Food Safety Authority (EFSA) Panel on Dietetic Products, Nutrition and Allergies (NDA), pursuant to Regulation (EC) No. 258/97, announced that novel food supplements from phlorotannins (marketed as SeaPolynolTM) are safe for human consumption [57].

In the food industry, the carrageenan application is regulated by the Commission Regulation (EU) No 231/2012 in Europe, which states that commercial carrageenan (E 407) fundamentally involves potassium, sodium, magnesium, and calcium sulphate esters of galactose and 3,6-anhydrogalactose polysaccharides [128]. The commercial form of agar approved for food industry by the Food and Drugs Administration (FDA) and the European Food Safety Agency (EFSA), in Europe is coded E-406 by the Commission Regulation No 257/2010 [129].

Agar is widely employed in biotechnological and research sectors, for example to produce biological culture media. However, low-quality agar is used in food products such as candies, fruit juice, frozen foods, bakery icing, or meringues [13]. *Porphyra/Pyropia* spp., *Eucheuma* spp., *Kappaphycus alvarezii*, and *Gracilaria* spp. (Rhodophyta) are the species most cultivated for exploitation of hydrocolloid for food industry [95].

The λ-carrageenan and κ-carregeenan of fam. Gigartinaceae are special type of carrageenan which are in the market as they generate quality and exhibit high viscosity in drinks, such as milk with chocolate [73]. The species most exploited for agar extractions belong to the genera *Gelidium*, *Gracilaria* and *Pterocladiella* [130].

Red algae are also rich in minerals and vitamins useful for our health. *Palmaria palmata* (Rhodophyta) contains high values of vitamin C that facilitates the absorption of iron, phycoerythrin a precursor to vitamin A, and trace of iron, potassium, and iodine that represent the 30% of its dry weight, while the 18% of the weight represents proteins of high nutritional value [131]. Another advantage of red algae assumption is the uptake of calcium for our organisms. It has been analysed the presence of calcium in *Chondrus crispus* (Rhodophyta), commonly known as Irish moss, and compared it with the calcium concentration present in the milk, which was lower [132].

Chlorophyta has been used as food source since long time. *Caulerpa* spp. is consumed as “seagrape” while *Ulva* spp. is consumed as whole food in salads [103]. Bioactive compounds of green seaweeds possess antioxidant, anticoagulant, antimutagenic, antibacterial, and anticancer activities [133]; thus, they have the potential to be functional foods. *Ulva lactuca* (Chlorophyta) extracts showed antimicrobial and photocatalytic activities [106]. The polysaccharide ulvan has been developed as a vegan alternative to beef-derived gelatine [134]. *Caulerpa* spp., especially *Caulerpa racemosa* (Chlorophyta), was found to be rich in phenolic and flavonoid compounds such as cyanidin, malvidin, quercetin, kaempferol, and apigenin. These metabolites are responsible for the high biochemical (antioxidant, scavenging, and reducing) and anti-proliferative activities of cancer line cells. Moreover, nutritional antioxidants and metabolites, make *Caulerpa racemosa* a promising functional food [102].

### 3.2. Biomedical Applications

Seaweeds bioactive compounds possess properties which make them attractive for biomedical applications.

Long time before the scientific research knowledge, many species of seaweeds have been used in traditional medicine, especially in Asian countries against goitre, nephritic diseases, anthelmintic, catarrh, just to new few diseases as medicaments or as pharmaceutical auxiliaries [135]. Among brown seaweeds *Laminaria* spp. was employed mainly in Japanese folk medicine for lowering the blood pressure, while *Fucus vesiculosus* has been used as a medicinal drug, mainly on account of its iodine content, for obesity defects and goitre [135], for the treatment of sore knees [136], healing wounds [137] and also as herbal teas for their laxative effects [138].

Carrageens of *Chondrus crispus* (Rhodophyta) have been used for a long time as a medicinal drug as a mucilage against diarrhoea, dysentery, gastric ulcers and as a component of numerous health teas, for example, for colds. *Gelidium cartilagineum* (Rhodophyta) has been used in Japan for colds and scrofula in paediatric medical science [135]. *Ulva lactuca* (Chlorophyta) has been used in folk medicine for gout and as astringent [135].

Extracts of Rhodophyta are very promising natural compounds to be exploited in biomedicine. Many species of Asian seaweeds are used as traditional medicine, such as *Gracilaria* spp. (Rhodophyta) which are used as laxative, *Sargassum* spp. (Phaeophyceae) are used to cure Chinese influence, while *Caloglossa* spp., *Codium* spp., *Dermonema* spp. and *Hypnea* spp. (Rhodophyta) are used as vermifuge drugs [139].

The biological activities of carrageenans make these compounds potential candidates for new antitumoral therapies, due to their antitumor immunity activation [7]. For example, *Kappaphycus* species (Rhodophyta) are used to reduce ulcers, headaches and the incidence of tumours [139]. κ-carrageenans isolated from *Kappaphycus striatum* demonstrated antitumoral activity against human nasopharynx carcinoma, human gastric carcinoma, and cervical cancer cell lines [94]. Different species belonging to the genus *Laurencia* (Rhodophyta) were tested to verify the bioactivity of their compounds. Some halogenated metabolites of *Laurencia papillosa* showed in vitro activity against human tumour cells Jurkat (acute lymphoblastic leukemia) [99]. Extracts of *Laurencia obtuse*, particularly in three different sesquiterpenes, have been isolated and evaluated against Ehrlich ascites carcinoma cells. The results showed antitumoral activity of the sesquiterpenes against Ehrlich ascites cells [140]. Ethanol extracts of *Gracilaria edulis* showed anticancer activity against ascites tumors in mice [97].

*Undaria pinnatifida* (Phaeophyceae) have anti-inflammatory properties that can be used as medicine to women after childbirth. This alga can also be used to lower fever, cure edema, and as a diuretic. Celikler et al. [107] surveyed the in vitro antigenotoxic activity in human lymphocytes from extracts of *Ulva rigida* (Chlorophyta). Although the antigenotoxic activity itself is not accentuated, these extracts possess strong protective effects against chemotherapeutic agent mitomycine-C.

During the past decade, studies suggested the use of seaweeds to prevent neurogenerative diseases [141]. The most common are Alzheimer’s disease (AD), Parkinson’s disease, Huntington’s disease, and Amyotrophic Lateral Sclerosis (ALS) [142].

Several studies highlight the use of algal polysaccharides for the treatment of neurodegenerative diseases as referred by Bauer et al. [143]. Park et al. [144] demonstrated improved memory and learning in mice treated with fucoidan extracts from *Ecklonia cava*; thus, the study suggests promising results in future human trials [144]. Mice treated with polysaccharide extracted from *Sargassum fusiforme* also showed improved memory and improved cognition, compared to the control group [145]. Phlorotannins of *Ecklonia cava*, in particular dieckol and phlorofucofuroeckol, are related with the increment of major central neurotransmitters in the brain, particularly of Acetylcholine (ACh) [146]. *Eisenia bicyclis* phlorotannins have been studied by Ahn et al. [147]; the authors demonstrated that 7-phloroeckol and phlorofucofuroeckol A were potent neuroprotective agents against induced cytotoxicity, while eckol exhibited a weaker effect [147].

κ-carrageenans extracted from red algae have been also discovered can significantly reduce the rate of apoptosis induced by Aβ25-35 on SH-SY5Y cells, supporting the hypothesis κ-carrageenans possesses neuroprotective properties; thus, they could be exploited as potential therapeutic agent for the treatment of AD [148].

Sulphated polysaccharides isolated from the green algae *Codium fragile* have been recently investigated from Wang et al. [149] to verify the protection against hydrogen peroxide (H2O2)-induced damage in vitro and in vivo tests. The free radical scavenging activities were tested on monkey kidney fibroblasts (Vero cells) and on zebrafish embryos. Both in vivo and in vitro tests demonstrated the potential of polysaccharides extracted from *Codium fragile* as neurorepair in animals [149] (Table 5).

### 3.3. Pharmaceutical Applications

Seaweeds bioactive compounds are employed in pharmaceutical industry with the aim to contribute to new formulations for innovative drugs, in order to substitute synthetic compounds with natural ones. SPs from algae possess important pharmacological activities such as anticoagulant, antioxidant, antiproliferative, antitumoral, anti-inflammatory and antiviral activities [12,150,151] (Table 6).

Various SPs have been extracted from species from order Bryopsidales (Chlorophyta) and Dictyotales and Fucales (Phaeophyceae) showed anticoagulant activity similar to heparin due to the in vitro inhibition of factors Xa and IIa mediated by antithrombin and heparin cofactor II [30]. Both in vitro and in vivo experiments demonstrated that fucoidans extracted from *Laminaria cichorioides* (Phaeophyceae) [92] and *Fucus evanescens* [93] behave like heparin as well; thus, they show anticoagulant activity accelerating the development of antithrombin III to inhibit the effect against thrombin.

Moreover, the APTT test, which measures the Activated Partial Thromboplastin Clotting Time, has been performed with fucoidans extracted from *Fucus vesiculosus* and demonstrated that the clotting time was higher under the influence of these natural compounds in respect to the control. Additionally, the TT test (Thrombin Time), a blood test that measures the time it takes for a fibrin clot to form in the plasma of a blood sample, revealed a higher clotting time respect the control as well [152].

Fucoidans exert several properties. For example, Pozharitskaya et al. [152] evaluated the antioxidant, anti-inflammatory, anti-hyperglycaemic and anticoagulant bioactivities of high-molecular-weight fucoidans extracted from *Fucus vesiculosus*. These compounds showed free-radical scavenging activity, even thought was lower than synthetic antioxidants, but its activity is comparable to the natural antioxidant showed from the natural antioxidant quercetin, present in plants [152]. Moreover, the inhibitory activity has been shown on both isoforms of the pro-inflammatory cyclooxygenase (COX-1 and 2) enzymes, considering fucoidans extracted from *Fucus vesiculosus* promising compounds for anti-inflammatory natural drugs [152]. Fucoidans from *Fucus vesiculosus* are also involved in the inhibition of the enzyme DPP-IV by fucoidan. This enzyme is related with the degradation of incretin hormones, which prevents higher amount of glucose in the blood (postprandial hyperglycemia); the new pharmaceutical is developing new DPP-IV inhibitors in order to decrease the glucose level in the blood and assure the anti-hyperglycaemic effect. Thus, the research of Pozharitskaya et al. [152] assesses that fucoidans may be involved in anti-hyperglycaemic activity trough the inhibition of DPP-IV.

Previous studies have reported that *Sargassum fulvellum* (Phaeophyceae) contains many bioactive molecules, such as phlorotannins, grasshopper ketone, fucoidan, and polysaccharides. *Sargassum fulvellum* extracts have been studied for years for analysing their diverse pharmacological effects such as antioxidant, anticancer, anti-inflammatory, antibacterial, and anticoagulant activities [153,154]. Extracts of *Sargassum fulvellum* were analysed to treat diseases such as a lumps, swelling, testicular pains, and urinary tract infections [153,155,156].

Agar extracted from red algae is widely used in biomedicine as suspension component in drug solutions and in prescription products, but also as anticoagulant agents and as laxative in capsules [13]. Due to its biological and pharmacological properties, the red algae *Gracilaria edulis* is known all over the world. Extract of *Gracilaria edulis* showed diverse properties such as antidiabetic, antioxidant, antimicrobial, anticoagulant, anti-inflammatory, and antiproliferative activities [96]; thus, these compounds are apposable for new pharmaceutical formulations. Furthermore, Gunathilaka et al. [98], evaluated the in vitro hypoglycemic activity of phenolic, flavonoid and alkaloid extracts from *Gracilaria edulis*. The study revealed the hypoglycaemic potential of the red alga through the inhibition of carbohydrate-digesting enzymes, glucose absorption, and the formation of antiglycation end products. *Ulva rigida* (Chlorophyta) has been reported hypoglycemic effect in vivo as well [108].

The antiviral properties of seaweeds make them another valuable choice to ameliorate the health of infected people; moreover, their application in pharmaceutical will ensure new and natural antiviral agents, which can substitute synthetic compounds. Additionally, the implication of seaweeds’ bioactive compounds is cost-efficient compared to the production of synthetic antivirals [157]. It has been discovered the protective effect of macroalgae antiviral activity against several viruses, such as human immune deficiency virus (HIV), Herpes Simplex Virus (HSV), genital warts [5], hepatitis C (HCV) [158]. Chlorophyta species have been proven effective against HSV [159,160], Encephalomyocarditis virus, Influenza “A” virus [161] or human metapneumovirus [110], to name a few. The antiviral action of macroalgae was first described by Gerber et al. [162] and it is associated with several compounds as fatty acids, diterpenes, but mainly with the presence of SPs [2,150,162,163], which can inhibit replication of viruses or can contribute to improve the immune system to fight against the viral infection. The protein griffithsin found in red algae *Griffithsia* sp. (Rhodophyta) showed antiviral activity against MERS-CoV virus [100] and SARS-CoV glycoprotein [101]. It is likely that these compounds act against SARS-CoV-2. Seaweeds’ polysaccharides can contribute for the development of powerful antivirals, indeed polysaccharides extracted from *Saccharina japonica* (Phaeophyceae) demonstrate in vitro inhibition to SARS-CoV-2 [164].

Even though the development of new antiviral formulations to cure viral infections is a real possibility [165], it is necessary to implement the evaluation and research of algal bio-compounds: only through the screen of high number of bioactive compounds might be possible to develop new natural drugs.

### 3.4. Cosmetics

Extracts of vitamins, minerals, amino acids, sugars, lipids and other biologically active compounds of several species of red and brown seaweeds are involved in cosmetic industry [166]. For instance, *Macrocystis pyrifera* (Phaeophyceae) commonly known as giant kelp, possesses phycocolloids that are used as a thickening agent in cosmetics by other industries [167].

Phloroeckol and tetrameric phloroglucinol (phlorotannins) from *Macrocystis pyrifera* revealed to exhibit antioxidant activity, which can contribute to prevent skin aging [90], thus protecting the skin from exposure to UV radiation and delaying the natural physiological changes; it also provides the consumer with a better quality of life and improves self-esteem [168].

Extracts of *Fucus vesiculosus* (Phaeophyceae) have been used to reduce the appearance of “eye bags” and the dark circles on the skin area under the eyes. The anti-inflammatory and antioxidant properties of the extracts stimulate the expression of haem oxygenase-l (HO-l), a molecule that eliminates the haem production on the skin by removing haem catabolites. Furthermore, this extract stimulates collagen production that could help to reduce fine lines and wrinkles. In addition, it could diminish or even avoid skin aging by using make-up and sunscreens [91].

Rhodophyta bioactive compounds are widely used in cosmetics, especially iota-carrageenan and κ-carrageenan which are available in products in the market. Creams, lotions, toothpaste, hair tonics, soaps, sunscreens, etc., are frequently enriched with carrageenan [169].

Bioactive compounds from Chlorophyta are also included in cosmetic industry. The antioxidant activity of the polysaccharide ulvan is attractive for novel and natural cosmetic formulations, since it was demonstrated, in vitro*,* the protective capability against hydrogen peroxide-induced oxidative stress. Furthermore, the presence of glucuronic acid adds moisturizing properties, and the rhamnosyl residues assure cell proliferation and collagen synthesis capacities, confirming ulvan as interesting raw material for the cosmetic industries [170].

## 4. Seaweeds Extracts in Industrial Applications

Seaweeds biological compounds are widely exploited in several industrial applications (Table 4). For example, these compounds have been explored for the production of biogas and biodiesel, which can be an alternative and efficient fuel to replace the use of fossil fuels. The SP ulvan extracted from the green seaweed possesses attractive physicochemical properties and biological activities, resulting in its applications in different innovative applications [171,172].

Ulvan extracted from *Ulva lactuca* (Chlorophyta) has been tested for production of biogas [104] and biodiesel [105]. Moreover, the optical, structural, thermal, and antioxidant properties make ulvan a potential contribute for new packaging material for food [173]. Ulvan from *Ulva fasciata* (Chlorophyta) was extracted and utilized to create edible films for food application. The films presented good mechanical and physicochemical properties adapted for containing food. The water vapour permeability in the pack decreased, preserving better the food. Moreover, ulvan from *Ulva fasciata* presents strong antioxidant activity [109], making this polysaccharide a perfect candidate for the production of novel, sustainable and eco-friendly bioplastics.

### 4.1. Agriculture

Seaweeds are utilized as compost to improve soil fertility and ameliorate plants cultivations [15,16,17]. The use of seaweeds as organic fertilizer in agriculture compensates the deficiency of plant nutrients such as nitrogen, phosphorous and potassium [174].

However, to avoid excess of salt, sand, and heavy metal content into the soil by using the whole seaweed [175], extracts of seaweeds have been considered for production of fertilizers [16,176,177]. Many countries already utilized seaweeds extracts to produce new formulations for fertilizers in agriculture [15,178] because of their advantages which are: stimulation of seed germination, improved crop performance and yield, increased resistance to abiotic and biotic stress such as phytopathogenic fungi [179], bacteria [180], insects [181] and enhanced postharvest life and quality of the product [16,182]. Moreover, the use of seaweed is a solution to overcome hazards caused by the extensive use of chemical fertilizers. Chemical fertilizers can give us health issues, while seaweed derived fertilizers are biodegradable, non-toxic, non-polluting and non-hazardous to human beings, animals and birds [183].

It is well known that macro and microminerals are essential to soil fertility and plant development; thus, the involved in seaweeds rich in minerals and nutrients could be a sustainable solution. The brown algae *Saccharina japonica* contains Ca, Mg, P, K, and Na as the main macrominerals, and Fe, I, Mn, Zn, and Al as the principal microminerals [184], therefore its extracts can contribute for the formulation of new biofertilizers. The European algae *Saccorhiza polyschides* (Phaeophyceae) has been evaluated from Soares et al. [174]. The study demonstrated that its minerals and essential trace elements were present in high quantity, suggesting *Saccorhiza polyschides* an interesting candidate for biofertilizer.

In recent years, the consideration for spray fertilizers based on seaweed increases, and they are extensively used in agriculture and horticulture [185]. Seaweed liquid fertilizer of extract of *Codium decorticatum* (Chlorophyta) has been tested to improve the growth of chilly plants. The effect of N, Mg, K and some trace elements contained in the extracts were positive for the crop plants. Extracts of *Codium decorticatum* were also used as biostimulants of tomato seed germination and plant growth. It was found that the compounds in the extract from green seaweeds increased seed germination percentage, plant biomass, and the content of chlorophylls a and b [186]. Thus, the liquid fertilizer from *Codium decorticatum* might be a solid and low-cost biofertilizer [187].

Considering the new approach of the agricultural industry, where the search of new sustainable and eco-friendly products is increasing, the role of seaweeds is extremely important. The utilization of natural fertilizers assures soils conditioned by natural antifungal agents [188] and healthy crops. Therefore, the involvement of seaweeds in agriculture will contribute also for human health welfare.

### 4.2. Animal Feed

The addition of seaweeds extracts in feed products will reduce the use of synthetic substances, which can provoke health issues. The demand for natural food or natural supplement food is growing. Unfortunately, the food safety regulatory framework is not fully harmonized between the countries, creating a problem in feed safety chain, increasing the animal health risks; consequently, human health is at risk when consuming animal meat [189]. Thus, the inclusion of seaweeds in animal diets will improve the quality of food and ensure sustainable and safe products.

Seaweeds are already used to enrich animal diets, for example, extracts obtained from *Ascophyllum nodosum* (Phaeophyceae) in Norway and the UK, *Laminaria digitata* (Phaeophyceae) in France, *Ascophyllum* and *Laminaria* species in Iceland already provide the main commercial ingredients used to feed land animals [190]. *Ascophyllum nodosum* extracts can be promising additive to increase food safety. These extracts have been mixed at 2% in meal for cattle and lambs during the 2 last weeks before slaughter. A reduction in *Escherichia coli* O157:H7 has been reported [191,192,193], possibly due to the presence of phlorotannins [194]. Reduction in other pathogenic microorganism such as *Salmonella* sp., *Campylobacter* species and *Clostridia* in the gastrointestinal track of domestic animals has also been observed [195].

The use of seaweed might be a solution of uptake of iodine in cases of iodine deficiency intake. *Ascophyllum nodosum* has been used to feed pigs in Belgium, by integrating 2% dried seaweed (the seaweed-based diet contained 10 mg/kg of iodine vs. 1 mg/kg for the control diet) into pig feed. The iodine concentration increases from 2.7 to 6.8, in different tissues. This feeding strategy for producing iodine-enriched meat was found to be a good solution to human iodine supply, without risk for overdosing or the need for a shift in eating pattern [196].

Additionally, seaweed extracts utilized as supplement to fish diets enhance the growth, lipid metabolism, physiological activity, stress response, disease resistance and carcass quality of various fish species [197,198,199]. For example, *Saccharina latissima* (Phaeophyceae) demonstrated potential as feed additive: its bioactive compounds ameliorate fish farming, and also increase protective activity against oxidative stress in fish [200].

## 5. Conclusions

Marine seaweeds are a great food source with bioactive components that promote a healthy diet with the advantage to exhibit anticancer, antiviral, antifungal, antidiabetic, antihypertensive, immunomodulatory, anticoagulant, anti-inflammatory, antioxidant, UV-protective, and neuroprotective properties after assimilation. Their low amount of fat makes them a key food in dietary. Brown seaweeds are among the most exploited, with red algae, widely used in food, nutraceutical, pharmaceutical and cosmetic industry. Unfortunately, not all seaweeds follow the criteria to be considered as food within safety standards. Seaweeds tend to accumulate heavy metals and minerals, which could deteriorate animal and human health if consumed in food or drugs.

The use of seaweeds bioactive compounds in biotechnological and industrial applications will promote a healthier lifestyle in a sustainable way. Thus, it is important to sustain and go further on the research of bioactive compounds of seaweeds in order to recognize and neutralize the effect of potentially harmful compounds, and increase the application of seaweeds’ compounds. The use of seaweeds bioactive compounds in biotechnological and industrial applications will promote a human well-being by contributing for the usage of natural products instead of chemicals.

## Figures and Tables

**Table 1:** Biological compounds isolated from Phaeophyceae.

Type of Algae	Isolated Compounds	Type of Compound	Reference
Phaeophyceae	Laminaran	Polysaccharide of glucose	[26,41,42]
	Fucoidan	Sulphated polysaccharide	[28,29,30,31]
	Alginic acid	Polysaccharide	[38,39]
	Phlorotannin	Polyphenolic compound	[48,49,50,51,52]
	Fucoxanthin	Pigment	[22,23,24,25]

**Table 2:** Biological compounds isolated from Rhodophyta.

Type of Algae	Isolated Compounds	Type of Compound	Reference
Rhodophyta	Carrageenans Agar	Sulphated polysaccharides	[7,65,66]
	Sesquiterpenes	Mixture of polysaccharide agarose and small molecules Terpenes	[69,70,71,72]
	Diterpenes	Terpenes	[63,64]
	Triterpenes	Terpenes	[63,64]

**Table 3:** Biological compounds isolated from Chlorophyta.

Type of Algae	Isolated Compounds	Type of Compound	Reference
Chlorophyta	Ulvan	Sulphated polysaccharides	[87,88,89]
	Palmitic acid	Saturated fatty acid	[87,88,89]
	Linoleic acid	Polyunsaturated fatty acid	[78,80]
	Chlorophylls (a and b)	Pigments	[77]
	Carotenoids (β-carotene and xanthophylls)	Pigments	[77]

**Table 4:** Main compounds of seaweeds involved in biotechnological applications.

Seaweed	Main Bioactive Compound	Property	Biotechnological Application	Reference
Phaeaophyceae				
*Laminaria hyperborea*	Alginate	Biodegradability, biocompatibility, non-toxic behaviour	Cosmetics, pharmaceutical and food industries as stabilizers	[18,40]
*Ascophyllum nodosum*				
*Ecklonia radiata*				
*Durvillaea* sp.				
*Lessonia* sp.				
*Sargassum* sp.				
*Scytothalia dorycarpa*				
*Cystophora subfarcinata*				
*Sargassum linearifolium*				
*Macrocystis pyrifera*	Alginate	Biodegradability, biocompatibility, non-toxic behaviour	Cosmetics as a thickening agent	[36]
	Phlorotannins	Antioxidant activity	Cosmetics for preventing skin aging	[90]
*Ecklonia cava*	Phlorotannins	Anticancer, antioxidant, anti-inflammatory, antiviral activities and antihypertensive effects.	Pharmaceutical and nutraceutical industries	[49,50,53]
*Eisenia arborea*	Phlorotannins	Antiallergic effects	Pharmaceutical industry	[62]
*Eisenia bicyclis*	Phlorotannins	Antidiabetic, antioxidant, antitumor, anti-inflammatory, and anticancer activities	Pharmaceutical and medical industries	[61]
*Ecklonia kurome*				
*Ecklonia stolonifera*				
*Pelvetia siliquosa*				
*Ishige okamurae*				
*Fucus vesiculosus*	Phlorotannins	Anti-inflammatory and antioxidant properties	Cosmetics, to produce make-up and sunscreens	[91]
*Fucus evanescens*	Fucoidans	Anticoagulant activity	Potential substitute to heparin	[92,93]
*Laminaria cichorioides*				
Rhodophyta				
*Chondrus pinnulatus*	λ-carrageenan and κ-carrageenan	High viscosity in drinks; antitumoral property	Food industry (production of drinks, e.g., milk and chocolate) and pharmaceutical industry	[7,73]
*Chondrus armatus*				
*Chondrus yendoi*				
*Kappaphycus striatum*	κ-carrageenan	Antitumoral activity against human nasopharynx carcinoma, human gastric carcinoma, and cervical cancer cell lines	Pharmaceutical industry	[94]
*Kappaphycus alvarezii*	κ-carrageenan and agar	Antioxidant properties	Cosmetics and nutraceutical industry	[68,95]
*Gracilaria edulis*	Agar Phenolic, flavonoid, and alkaloid compounds	Antidiabetic, antioxidant, antimicrobial, anticoagulant, anti-inflammatory, and antitumoral activities; hypoglycaemic activity	Pharmaceutical industry	[74,96,97,98]
*Laurencia catarinensis*	Halogenated metabolites	Antitumoral activity	Pharmaceutical industry	[99]
*Laurencia obtuse*	Diterpene and sesquiterpene	Actions against different cancer cell lines (KB, HepG2 and MCF-7)	Pharmaceutical industry	[99]
*Griffithsia* sp.	Griffith (Protein)	Antiviral activity against MERS-CoV-2 virus and SARS-CoV-2 glycoprotein	Pharmaceutical industry	[100,101]
Chlorophyta				
*Caulerpa racemosa*	Phenolic compounds and flavonoids	Antioxidant, scavenging, anti-proliferative activities of cancer line cells	Pharmaceutical and nutraceutical industries	[102,103]
*Ulva lactuca*	Ulvan	Antioxidant activity, antimicrobial and photocatalytic activities	Food industry (the whole body is used as salad) and industrial industry (production of biogas and biodiesel)	[83,103,104,105,106]
*Ulva rigida*	Ulvan	Antigenotoxic activity in human lymphocytes; hypoglycaemic effect in vivo experiment	Pharmaceutical industry	[107,108]
*Ulva fasciata*	Ulvan	Antioxidant and good mechanical properties; antiviral property	Industrial industry to develop bioplastics; pharmaceutical industry	[109,110]

**Table 5:** Preclinical studies on cell lines performed with seaweed bioactive compounds.

Preclinical Trial	Cell Lines Surveyed	Dosage (µg/mL)	Effect	Reference
Antitumoral activity of carregaagenans and oligosaccharide fractions of carregaagenans from *Kappaphycus striatum*	Human nasopharyngeal carcinoma (KB), human gastric carcinoma (BGC) and human hela carcinoma (Hela)	500, 250, 125	The results of bioassay showed that the fraction F1 exhibits relatively higher antitumor activity against three cancer cells in vitro than polysaccharides	[94]
Antitumoral activity of ethanol:water extracts and ethanol:chloroform extracts of *Laurencia papillosa*	Jurkat cancer cells	25–1000	The number of the viable cells is decreased with ethanol:chloroform extract with IC50 value of 57.77 µg/mL is (more cytotoxic than the ethanol:water extract with IC50 value of 121.642 µg/mL)	[99]
Antitumoral activity of three sesquiterpenes (12-hydroxy isolaurene 8,11-dihydro-12-hydroxy isolaurene and isolauraldehyde) obtained from extract of the red alga *Laurencia obtusa.*	Ehrlich cells (Ehrlich ascites Carcinoma, EAC)	25, 50, 100	Isolauraldehyde proved to have the highest cytotoxic activity (83.1%) followed by compound 2 (79.9%)	[140]
Antitumoral activity of ethanolic extract of *Gracilaria edulis*	Ehrlich ascites tumour (EAT) cells from mice	0–100	EAT cells viability was close to 65% At 50 μg/mL dose and the maximum decrease of 15% was observed at 100 μg/mL	[97]
Antigenotoxicity activity of *Ulva rigida* crude extracts on human lymphocytes and protective effects on chemotherapeutic agent mitomycine-C.	*In vitro* human lymphocytes	10, 20, 40	Genotoxic activity in human lymphocyte cell culture was not high, while *Ulva ridiga* extracts significantly decreased the number of chromosomal aberrations, the frequencies of sister chromatid exchange and micronuclei, compared with the cell culture treated with chemotherapeutic agent mitomycine-C	[107]
Activation of LXRα or LXRβ (nuclear receptor) from polysaccharide extracts of *Sargassum fusiforme*	Human microglia cells (CHME3) from University Paris-Sud, France and in vivo from mice used as model of survey for AD	1, 3, 5	In vitro CHME3 cells showed a significantly activation of LXRβ but not LXRα with dose of 5 µg/mL. In vivo test showed after ten weeks LXR activation in the central nervous system, evidenced by a cerebral induction of LXR response genes	[145]
Protection against Aβ- induced neurotoxicity in PC12 cells trough isolated phlorotannins from *Eisenia bicyclis*	Rat pheochromocytoma cells (PC12 cells) obtained from American Type Culture Collection (ATCC)	2.5, 5, 10, 20	7-phloroeckol and phlorofucofuroeckol A have been shown to be potent neuroprotective agents	[147]
Protection against hydrogen peroxide (H2O2)-induced damage trough sulfated polysaccharides from *Codium fragile*	Monkey kidney fibroblasts (Vero cells) Zebrafish embyos	12.5, 25, 50	In vivo and in vitro tests showed the potential of polysaccharides extracted as neurorepair in animals	[149]

**Table 6:** Pharmaceutical effects of seaweed bioactive compounds.

Seaweed	Compound Extracted	Cell Lines/Animals Surveyed	Route of Administration	Dosage (µg/mL)	Effect	Reference
*Laminaria cichorioides* (Phaeophyceae)	Sulphated fucan	Human plasma	The lyophilized crude polysaccharide was dissolved in human plasma	10, 30, 50	In vitro anticoagulant activity	[92]
*Fucus evanescens* (Phaeophyceae)	Fucoidans	Human plasma Rat plasma	Intravenous Injection	125, 250, 500, 1000	In vitro and in vivo anticoagulant activity	[93]
*Sargassum fulvellum* (Phaeophyceae)	Phlorotannins, grasshopper ketone, fucoidan and polysaccharides	Mice	Oral administration	Based on weight of mice	Antioxidant, anticancer, anti-inflammatory, antibacterial, and anticoagulant activities	[153]
*Gracilaria edulis* (Rhodophyceae)	Phenolic, Flavonoid and Alkaloid compounds	Bovine serum albumin (protein)	The extracts were tested on the protein	20, 40, 60, 80, 100, 120	Hypoglycaemic activity	[98]
*Ulva rigida* (Chlorophyceae)	Ethanolic extract	Twenty-four male Wistar rats	Oral administration	500 mL of water with extracts in 2% wt/vol as drinking water for exposed groups per each day (from 3 to 30 days).	In vivo anti-hyperglycaemic, antioxidative and genotoxic/antigenotoxic activities	[108]
*Griffithsia sp.* (Rhodophyceae)	Griffithsin (protein)	MERS-CoV and SARS-CoV glycoproteins	The extracts were tested on the proteins	0.125, 0.25, 0.5, 1, 2	Antiviral activity against MERS-CoV virus and SARS-CoV glycoprotein	[100]
*Saccharina japonica* (Phaeophyceae)	polysaccharides	SARS-CoV-2 S-protein	The extracts were tested on the protein	50–500	In vitro inhibition to SARS-CoV-2	[164]

## Data Availability

Not applicable.
